# Breast Abscess in Young Nulliparous Women: Is Injectable Breast Augmentation the Missing History?

**DOI:** 10.12669/pjms.42.8.16621

**Published:** 2026-08

**Authors:** Mifrah Rahat Khan Sherwani1, Safna Naozer Virji, Lubna Mushtaque Vohra

**Affiliations:** 1 Dr. Mifrah Rahat Khan Sherwani; 2 Dr. Safna Naozer Virji; 3 Dr. Lubna Mushtaque Vohra

In non-lactating women, breast abscess is uncommon but relatively well-documented in clinical practice. Among established risk factors, including smoking, diabetes mellitus, obesity, nipple piercings, and immunosuppression, Staphylococcus aureus and anaerobic flora are the most implicated. Physicians are trained to exclude inflammatory carcinoma, tuberculosis, and granulomatous mastitis in young nulliparous women presenting with the above conventional risk factors.^1^ Yet, the increasingly significant and unrecognizable etiology is, infection caused by previously injected breast filler material.

There is a surge of infections after the procedure of injectable breast fillers in unregulated and unsupervised clinics by noncertified bodies in Pakistan, and the problem is often complicated by the patients’ failures to disclose this history. The concealment of history at the time of surgical presentations is of course dangerous, but unfortunately commonplace. In our experience at a tertiary breast surgical hospital, several young women presenting with atypical, recurrent breast abscesses or treatment-resistant infections disclosed a previous history of injectable augmentation only upon repetitive and focused questioning and not at the first presentation of symptoms.

The prevailing sociocultural context is one of the reasons for this concealment. Elective breast augmentation carries a significant stigma in Pakistan’s largely conservative society, despite social media awareness campaigns and female empowerment.^2^ The concerns regarding stigma and family disapproval lead to reluctance to admit to these procedures performed in unregistered clinics by practitioners of uncertain qualification at a fraction of the cost in comparison to surgical breast augmentation.

The concealment of this can lead to serious clinical consequences. Infections associated with fillers, whether from polyacrylamide hydrogel (PAAG) or hyaluronic acid (HA), are sustained by foreign body nidus and cause infections that are ineffective with antibiotic monotherapy, especially if injected into the breast parenchyma rather than in the intended sub-glandular or subpectoral plane. This triggers chronic inflammatory foreign body reactions requiring surgical debridement for resolution.^3^ Nontuberculous mycobacteria, most notably Mycobacterium abscesses, have also been documented in post-procedural injectable fillers performed under inadequate sterile conditions and necessitate prolonged, culture-guided multi-antimicrobial therapy.^2^ Without knowledge of the filler history, none of these diagnoses is reasonably reached.

A single non-judgmental, timely asked question, “Have you ever had any injections or procedures done to your breasts for cosmetic purposes?” sensitively posed in a confidential encounter, may lead to the correct diagnosis, and can prevent further complications in our clinical practice. Furthermore, appropriate investigations such as microbiology cultures (including mycobacterial culture), ultrasound, and surgical management of debridement should be followed accordingly. Timely asking the right questions may save a breast, sometimes a life.
